# Addressing Multi-Center Variability in Radiomic Analysis: A Comparative Study of Image Acquisition Methods Across Two 3T MRI Scanners

**DOI:** 10.3390/diagnostics15040485

**Published:** 2025-02-17

**Authors:** Claudia Tocilă-Mătășel, Sorin Marian Dudea, Gheorghe Iana

**Affiliations:** 1 Department of Radiology, Faculty of Medicine, Iuliu Hatieganu University of Medicine and Pharmacy, 400012 Cluj-Napoca, Romania; sdudea@umfcluj.ro; 2 Medima Health SA, 060254 Bucharest, Romania; gheorghe.iana@medimahealth.ro

**Keywords:** radiomics reproducibility, multi-scanner variability, raw image acquisition

## Abstract

**Background**: Radiomics has become a valuable tool in medical imaging, but its clinical use is limited by data variability and a lack of reproducibility between centers. This study aims to assess the differences between two scanners and provide guidance on image acquisition methods to reduce variations between images obtained from different centers. **Methods**: This study utilized medical images obtained in two different imaging centers, with two different 3T MRI scanners. For each scanner, 3D T2 FLAIR sequences were acquired in two forms: the raw and the clinical practice images typically used in diagnostic workflows. The differences between images were analyzed regarding resolution, SNR, CNR, and radiomic features. To facilitate comparison, bias field correction was applied, and the data were standardized to the same scale using Z-score normalization. Descriptive and inferential statistical methods were used to analyze the data. **Results**: The results show that there are significant differences between centers. Filtering and zero-padding significantly influence the resolution, SNR, CNR values, and radiomics features. Applying Z-score normalization has resolved variations in features sensitive to scale differences, but features reflecting dispersion and extreme values remain significantly different between scanners. Some feature differences may be resolved by analyzing the raw images in both centers. **Conclusions**: Variations arise due to different acquisition parameters and the differing quality and sensitivity of the equipment. In multi-center studies, acquiring raw images and then applying standardized post-processing methods across all images can enhance the robustness of results. This approach minimizes technical differences, and preserves the integrity of the information, reflecting a more accurate representation of reality and contributing to more reliable and reproducible findings.

## 1. Introduction

Magnetic resonance imaging (MRI) is the primary imaging method for diagnosing and monitoring brain lesions. However, it can sometimes be challenging to distinguish between types of lesions, such as post-radiation necrosis and tumor progression, using standard MRI scans. Radiomics is an image analysis technique that applies advanced mathematics to reveal patterns in medical images that are not visible to the naked eye. Numerous studies have demonstrated that radiomics can differentiate between post-radiation necrosis and tumor progression. Although radiomic results are well documented, they are not currently used in clinical practice due to data variability between centers and a lack of reproducibility [1].

MRI images are primarily designed for visual interpretation, so techniques have been developed to enhance visual quality without adding factual information [1]. Filters and zero-padding are two such techniques.

Zero-padding in k-space involves adding zero values in the frequency domain to increase the apparent resolution in the resulting image without adding genuine information to the raw data.

Zero-padding in MRI imaging has both advantages and disadvantages. It enhances the apparent spatial resolution, making finer details more visible and aiding in diagnosis. It also reduces edge-related artifacts, leading to more uniform images.

However, zero-padding does not add new information to the raw data and can amplify noise, potentially degrading image quality.

Filters in MRI imaging enhance visual quality, increase the signal-to-noise ratio (SNR), and improve the contrast-to-noise ratio (CNR), which is essential for optimal lesion visualization. However, filters can introduce distortions, blur fine details, and produce variable results, leading to inconsistencies in image analysis [2].

Although many other acquisition parameters influence radiomic features, zero-padding and filtering are artificial interventions that do not reflect the true properties of tissue. Once applied during acquisition, filters and zero-padding become irreversible, and the original image cannot be fully recovered.

Using raw images provides a more accurate representation of reality, simplifies image processing, and reduces device-related biases. Implementing a standardized post-processing pipeline on raw data can result in more consistent and comparable outcomes in radiomic image analysis.

The purpose of this study is to assess the differences between two different scanners and provide guidance on image acquisition methods. This aims to minimize variations in images from different centers and enhance the robustness of radiomic results for future applications.

## 2. Materials and Methods

Two 3T MRI scanners were utilized: the General Electric (GE) Signa Pioneer (2018) (Boston, MA, USA), with a 21-channel phased-array head and neck coil, and the Siemens Healthineers Magnetom Lumina (2021) (Forchheim, Germany), with a 20-channel phased-array head and neck coil. For each scanner, 3D T2 FLAIR were acquired in two forms: the raw image, without filtering or zero-padding, and the clinical practice image typically used in diagnostic workflows.

A total of 28 patients were included in this study, with 14 patients scanned on each system. The inclusion criteria were established to ensure a homogeneous population in terms of brain structure, minimizing potential sources of variability. Only brain MRI examinations within normal limits, free of motion artifacts, and from individuals aged 18 to 60, without significant personal pathological history or uncontrolled systemic diseases, were included.

The exclusion criteria covered patients with intracerebral hemorrhages, brain tumors, demyelinating diseases, prior neurosurgical interventions, head trauma, and neurodegenerative diseases. Additionally, individuals with uncontrolled systemic diseases, systemic cancer, or any condition that could alter brain morphology or signal characteristics were excluded from this study.

The regions of interest analyzed to calculate SNR and CNR were the normal cerebral white matter at the level of the splenium of the corpus callosum and the gray matter at the level of the caudate nucleus. The splenium of the corpus callosum was selected for SNR calculation due to its stability, homogeneity, and consistency across different scanners and individuals, ensuring comparability. The segmentation of regions of interest was performed manually by a board-certified radiologist in their first year as a specialist.

The MRI images were converted from DICOM format to NIfTI (Neuroimaging Informatics Technology Initiative) using 3D Slicer. After loading the DICOM files into 3D Slicer, the images were exported as NIfTI (.nii). This conversion simplified data processing by preserving essential information, such as voxel dimensions and intensity, while eliminating unnecessary metadata [3]. Bias field correction was applied to reduce intensity inhomogeneities caused by variations in the magnetic field, thereby ensuring a uniform signal distribution. This was implemented in Python using the N4 Bias Field Correction algorithm from the SimpleITK library [4]. Z-score normalization was applied to the images to reduce scale differences, improving comparability and reliability in radiomic analysis. This was implemented in Python using the NumPy library [5]. Radiomic features were subsequently extracted from the splenium of the corpus callosum, a region chosen because it exhibits fewer anatomical variations between patients and scanners, providing a stable basis for image comparisons.

All data extractions were performed using 3D Slicer software (version 5.2.2), an opensource tool widely used for medical image processing [6].

For statistical analysis, we used descriptive statistics (percentage calculation) and inferential statistics, including the Shapiro–Wilk Test, Levene’s Test, ANOVA, the Kruskal–Wallis Test, the Post hoc Conover Test, the Post hoc Tukey Test, and Bonferroni correction [7]. All analyses were performed using Python (version 3.11.1) [8] with the following libraries: scipy.stats, statsmodels, and scikit_posthocs, and manual implementation for the Bonferroni correction.

## 3. Results

### 3.1. Resolution

For an accurate interpretation of radiomic features, high-resolution images are essential. The resolution of an image depends on the voxel dimensions: slice thickness, field of view (FOV), and matrix size.

**Figure 1:**
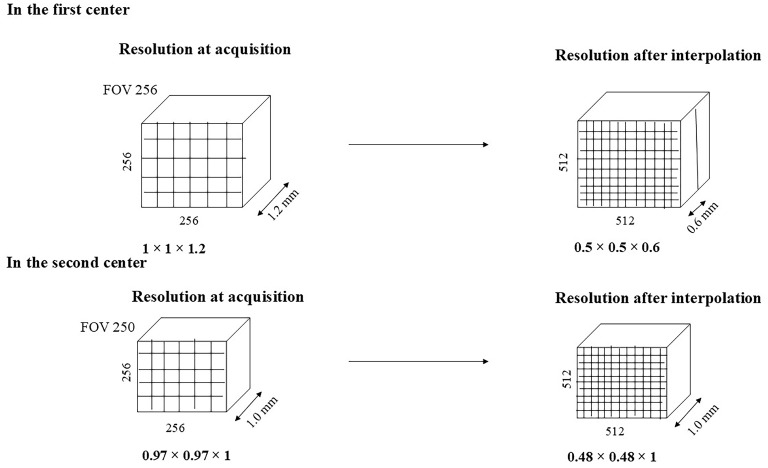
The effect of interpolation on image resolution.

The initial acquisition parameters in the first center included a FOV of 256, a matrix size of 256 × 256, and a slice thickness of 1.2 mm, resulting in a resolution of 1 × 1 × 1.2 mm. In clinical practice, to improve image resolution, zero-padding was applied both in-plane and through-plane, refining the matrix size to 512 × 512 and reducing the slice thickness to 0.6 mm, achieving a final resolution of 0.5 × 0.5 × 0.6 mm. In the second center, images were acquired with an FOV of 250, a matrix size of 256 × 256, and a slice thickness of 1 mm. In clinical practice, zero-padding was applied only in the matrix plane to enhance image resolution, resulting in a final resolution of 0.48 × 0.48 × 1 mm (Figure 1). If zero-padding had been applied to the slice thickness in the second center, it would have resulted in volumes much more comparable to those from the first center.

### 3.2. Signal-to-Noise Ratio (SNR)

Signal-to-noise ratio (SNR) is a measure used to evaluate the clarity of the signal relative to the noise level present in an image.

**Figure 2:**
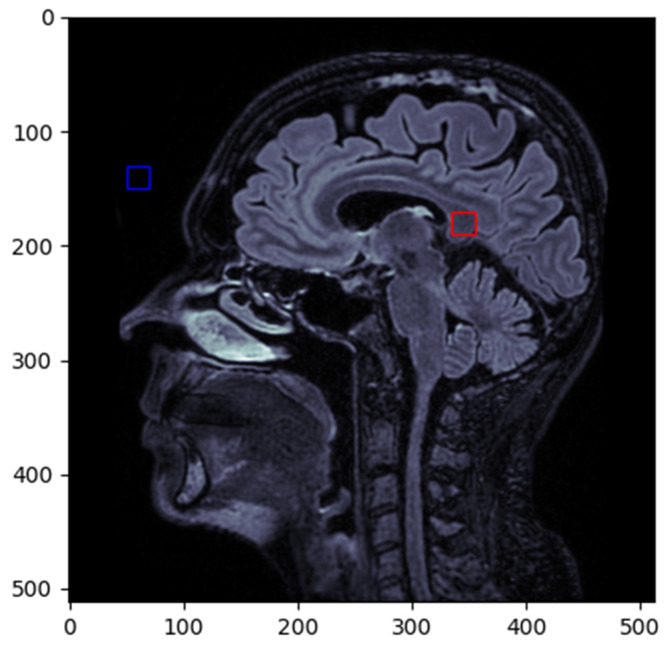
ROI selected for SNR calculation.

To calculate the SNR, we selected the splenium of the corpus callosum (red box) as the primary region of interest (ROI), and the noise was measured from the forehead region (blue box) (Figure 2).

A percentage formula highlighted the impact of zero-padding and filters on the SNR.

$$\frac{\mathrm{SNR}\;\mathrm{condition}-\mathrm{SNR}\;\mathrm{original}}{\mathrm{SNR}\;\mathrm{original}}\times 100$$

The original SNR was measured at 28.14 on average in the first center. Applying filters (F+) improved the SNR by 71.81%, increasing it to 48.35. Zero-padding alone (Z-padding) reduced the SNR by 6.04%, bringing it down to 26.44, while the combination of filters and zero-padding (F+ Z-padding+) led to an overall SNR improvement of 43.28%, reaching 40.32. In the second center, the original SNR was 56.08. Filters were not used in the second center, so no SNR improvement was calculated for this case. However, zero-padding alone caused a 10.32% decrease in SNR, reducing it to 50.29 (Table 1).

### 3.3. Contrast-to-Noise Ratio (CNR)

**Figure 3:**
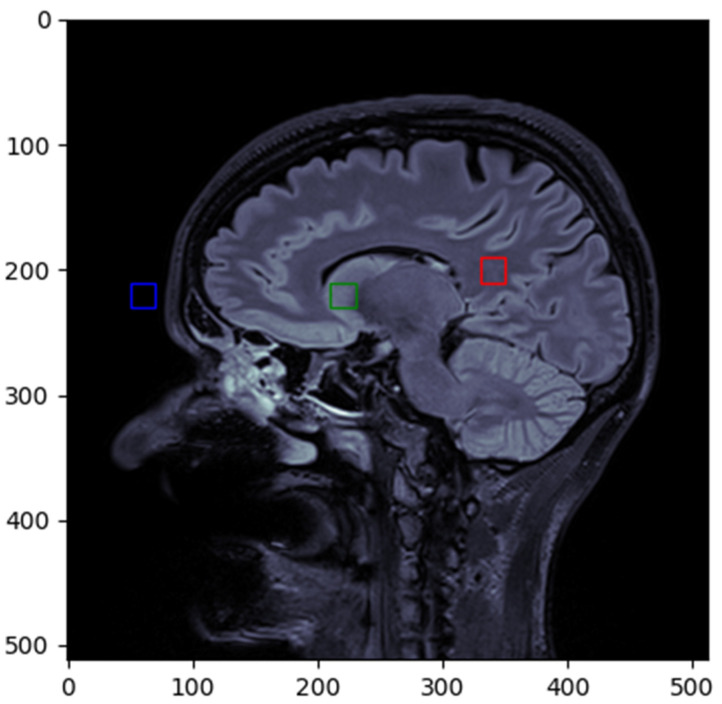
ROI selected for CNR calculation.

CNR (contrast-to-noise ratio) is a measure used to evaluate the quality of contrast between two structures or regions of interest, in relation to the level of noise present in the image. The contrast was measured between normal white matter (red box) and the gray matter at the level of the caudate nucleus (green box), while the noise was measured from the forehead region (blue box) (Figure 3).

A percentage formula highlighted the impact of zero-padding and filters on the CNR.

$$\frac{\mathrm{CNR}\;\mathrm{condition}-\mathrm{CNR}\;\mathrm{original}}{\mathrm{CNR}\;\mathrm{original}}\times 100$$

The original CNR was measured at 14.31 on average in the first center. Applying filters (F+) improved the CNR by 64.70%, increasing it to 23.57. Zero-padding alone (Z-padding) reduced the CNR by 0.34%, bringing it down to 14.26, while the combination of filters and zero-padding (F+ Z-padding+) led to an overall CNR improvement of 41.16%, reaching 20.20. In the second center, the original CNR averaged 22.83. Filters were not used in the second center, so no CNR improvement was calculated for this case. However, zero-padding alone caused a 9.15% decrease in CNR, reducing it to 20.74 (Table 2).

### 3.4. Radiomics

Radiomics involves extracting an extensive series of quantitative features from medical images. We analyzed both first-order features, referring to statistical features derived from the distribution of voxel intensities, and second-order features, also known as texture features. Out of 18 first-order features, 16 showed significant differences between scanners. Out of 75 s-order features, 69 showed significant differences between the scanners. To address these discrepancies, Z-score normalization was applied, bringing voxel intensities to a common scale with a mean of 0 and a standard deviation of 1, reducing scale differences and ensuring comparability between scanners.

#### 3.4.1. First-Order Features

Initially, out of 18 first-order features, 16 showed significant differences between scanners, except for Kurtosis and Skewness.

After applying Z-score normalization, 9 out of 18 first-order features no longer showed significant differences between scanners. However, nine features still exhibit significant variability between scanners, indicating residual inconsistencies despite normalization.

**Figure 4:**
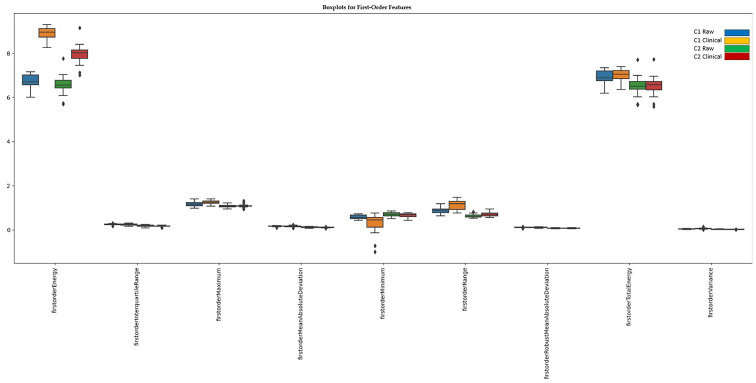
First-order features that still show significant differences between scanners after normalization.

The first two groups represent data extracted from the first center, including raw and clinical practice images. The last two groups represent data extracted from the second center, including raw and clinical practice images (Figure 4).

The features that have been resolved are related mainly to central tendencies (mean, median) and the concentration of extremes (percentiles).

The features that were not resolved after Z-score normalization include measures of dispersion (Interquartile Range, Range, variance), extreme values (Min, Max), and total energy distribution (total energy, first-order energy).

After normalization, two features (entropy and uniformity) were compressed to values between 0 and 1. This is likely due to the homogeneous region from which the data were extracted.

#### 3.4.2. Second-Order Features—GLCM (Gray Level Co-Occurrence Matrix)

Initially, out of 24 extracted GLCM features, 20 showed significant differences between scanners. After Z-score normalization, the values of all features were compressed to 0 or 1, indicating a loss of variability among the scanners.

#### 3.4.3. Second-Order Features—GLDM (Gray Level Dependence Matrix)

Initially, out of 14 extracted GLDM features, 12 exhibited significant differences, while 2 showed no significant differences between scanners: gldmLargeDependenceHighGrayLevelEmphasis and gldmSmallDependenceLowGrayLevelEmphasis. After Z-score normalization, nine features continued to show significant differences between scanners.

**Figure 5:**
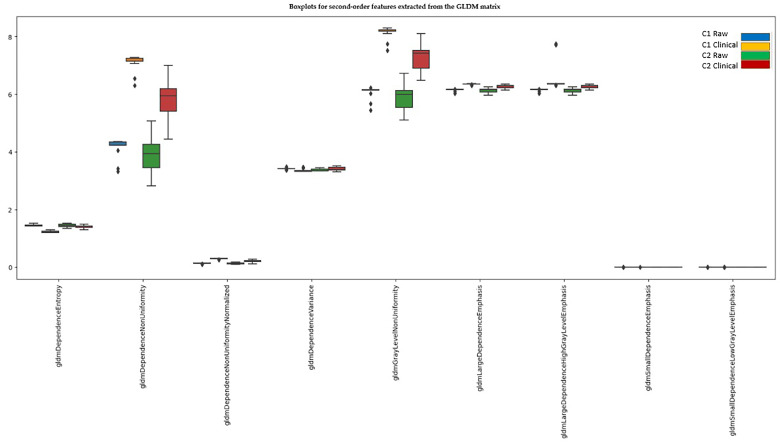
Second-order features extracted from the GLDM matrix that still show significant differences between scanners after normalization.

The first two groups represent data extracted from the first center, including raw images and clinical practice images. The last two groups represent data extracted from the second center, including raw images and clinical practice images (Figure 5).

After Z-score normalization, of the nine features showing significant differences, seven could potentially be resolved if the raw images are acquired in both centers. The features that are not dependent on scale and do not resolve after normalization are those related to nonuniformity.

Three features were compressed to 0 or 1 (gldmGrayLevelVariance, gldmHighGrayLevelEmphasis, gldmLowGrayLevelEmphasis).

Two features no longer showed significant differences (gldmLargeDependenceLowGrayLevelEmphasis, gldmSmallDependenceHighGrayLevelEmphasis).

#### 3.4.4. Second-Order Features—GLRLM (Gray Level Run Length Matrix)

Before normalization, all 16 extracted GLRLM features showed significant differences between scanners. After Z-score normalization, 10 features still maintained significant differences.

**Figure 6:**
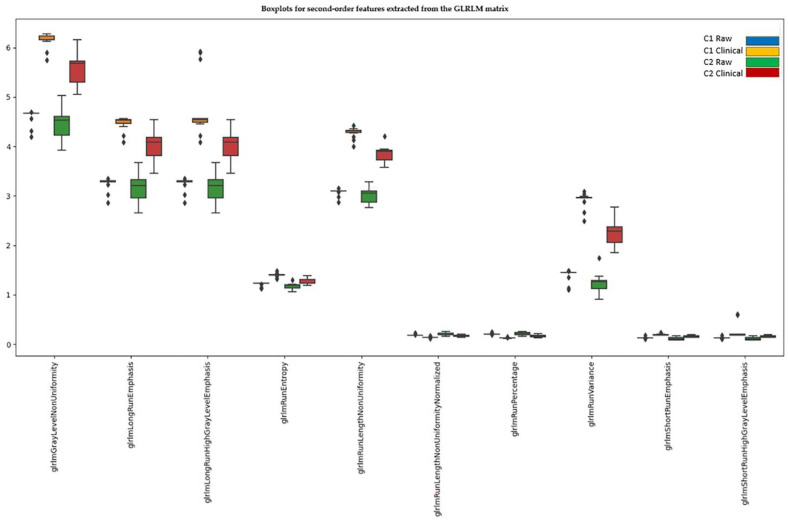
Second-order features extracted from the GLRLM matrix that still show significant differences between scanners after normalization.

Six features could potentially be resolved if the raw images are acquired on both scanners. The features that do not resolve after normalization are those related to nonuniformity, entropy, and variance (Figure 6).

Four features were compressed to 0 or 1 (glrlmGrayLevelNonUniformityNormalized, glrlmGrayLevelVariance, glrlmHighGrayLevelRunEmphasis, glrlmLowGrayLevelRunEmphasis), while two features showed no longer significant differences (glrlmLongRunLowGrayLevelEmphasis, glrlmShortRunLowGrayLevelEmphasis).

#### 3.4.5. Second-Order Features—GLSZM (Gray Level Size Zone Matrix)

**Figure 7:**
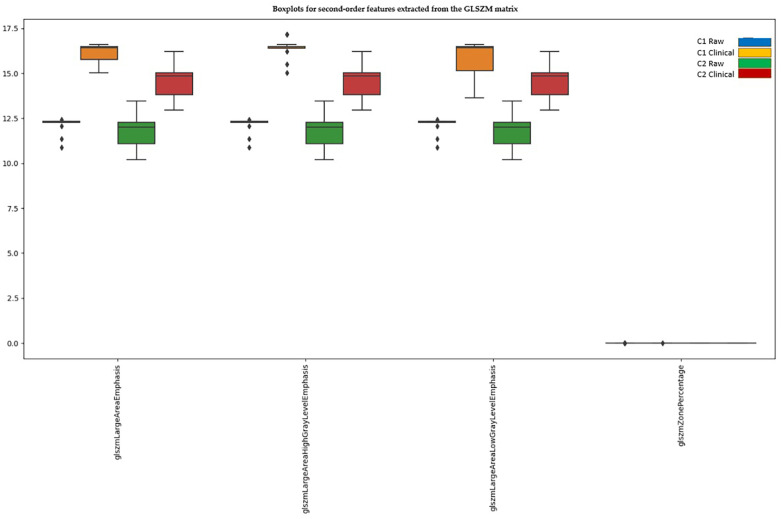
Second-order features extracted from the GLSZM matrix that still show significant differences between scanners after normalization.

In the case of GLSZM, out of 16 extracted characteristics, all exhibited significant differences between scanners before normalization. After Z-score normalization, four features maintained significant differences, while twelve features were compressed to 0 or 1 (Figure 7).

#### 3.4.6. Second-Order Features—NGTDM (Neighborhood Gray Tone Difference Matrix)

For NGTDM, all five extracted characteristics showed significant differences before normalization. After normalization, all characteristics were compressed to 0 or 1.

## 4. Discussion

Zero-padding for 2D imaging increases the “in-plane” matrix size (e.g., from 256 to 512 pixels). For 3D imaging, zero-padding can be used both “in-plane” and “through-plane”, affecting the slice thickness (e.g., from 64 to 128 slices) [2]. While it improves visual quality, it may also introduce distortions or artifacts, complicating interpretation, and automated algorithms may misinterpret the interpolated data. Additionally, it increases computational complexity and processing time, making image analysis more demanding [9,10].

Our study demonstrates that acquisition resolution and interpolation methods differ between the two centers. In the first center, zero-padding occurs both in the “in plane” affecting the matrix size and the “through plane” in the slice thickness, whereas, in the second center, interpolation is applied only “in plane”. The differences in zero-padding methods show the preferences of each center, being intrinsic to the acquisition protocols and reflecting routine clinical practice. This discrepancy can lead to inconsistencies between the scanner outputs, causing possible discordance when comparing the extracted radiomic features. The lack of standardization in acquisition workflows may limit the clinical applicability of radiomics, introducing artificial variability unrelated to biological differences.

SNR and CNR vary between the two centers and further illustrate the impact of acquisition settings on radiomic feature reliability. One center has inherently better SNR and CNR, while the other artificially achieves improved values by adding filters. This difference has direct implications on radiomic features. Low SNR introduces noise that distorts texture features like entropy and variance, while low CNR reduces the clarity of tissue boundaries, affecting features dependent on contrast, like contrast and homogeneity. This discrepancy may lead to potential misinterpretations of radiomic features.

Before normalization, most of the radiomic features showed significant differences between scanners. Z-score normalization was applied to bring the voxel intensities to a common scale to reduce intensity variations.

The application of Z-score normalization has resolved variations in features sensitive to scale differences (e.g., mean, median, skewness). However, features reflecting dispersion and extreme values remain significantly different between scanners. This indicates that normalization alone does not fully compensate for inter-scanner differences. Using raw images for analysis could potentially improve comparability for certain features. However, since variations arise not only due to different acquisition parameters, but also because of the equipment’s differing quality and sensitivity, additional harmonization techniques may be necessary.

After Z-score normalization, most radiomic features, particularly those extracted from GLCM, GLSZM, and NGTDM, were compressed to 0 or 1, likely due to the low variability within the homogeneous region of interest. This should be considered when analyzing highly uniform ROIs acquired from different scanners; as it may reduce interscanner differences, it could also obscure potentially significant radiomic features.

This study has several limitations that should be considered.

The use of two different 3T MRI scanners with distinct interpolation and filtering methods accounts for only a limited subset of the factors contributing to image variability between scanners. A broader range of acquisition parameters and scanner types, which were not considered in this study, also influence image differences.

The relatively small study population was also a limitation. Larger studies could provide more significant results.

The selection of homogeneous ROIs led to feature compression. This effect may not be generalizable to more heterogeneous tissues.

Although Z-score normalization reduced intensity-related variations, it did not fully harmonize radiomic features. More advanced harmonization techniques, such as deep learning-based approaches, were not explored in this study.

## 5. Conclusions

This study confirms that scanner-related differences, zero-padding, and filters affect radiomic feature consistency. While normalization helps, it does not eliminate variability, highlighting the need for standardized acquisition protocols.

Additionally, interventions such as zero-padding and filtering introduce extra variability. The use of raw images may offer potential advantages, including a more accurate representation of physiological reality, improved consistency in radiomic analysis, and a more streamlined image processing workflow. Future research should explore the benefits of raw image acquisition combined with advanced harmonization techniques to enhance the robustness and reproducibility of radiomic studies across different centers.

## Figures and Tables

**Table 1:** Impact of filters and zero-padding on SNR in two imaging centers.

	First Center	Second Center
F+	71.81%	-
Z-padding+	−6.04%	−10.23%
F+ Z-padding+	43.28%	-

**Table 2:** Impact of filters and zero-padding on CNR in two imaging centers.

	First Center	Second Center
F+	64.7%	-
Z-padding+	−0.34%	−9.15%
F+ Z-padding+	41.16%	-

## Data Availability

The data are not publicly available due to privacy and ethical restrictions.
